# Analysis of the Short- and Long-Term Immune Response in BALB/c Mice Immunized with Total *Naegleria fowleri* Extract Co-Administered with Cholera Toxin

**DOI:** 10.3390/tropicalmed11010022

**Published:** 2026-01-12

**Authors:** Mara Gutiérrez-Sánchez, Maria de la Luz Ortega-Juárez, María Maricela Carrasco-Yépez, Rubén Armando Herrera-Ceja, Itzel Berenice Rodríguez-Mera, Saúl Rojas-Hernández

**Affiliations:** 1 Laboratorio de Inmunobiología Molecular y Celular, Sección de Estudios de Posgrado e Investigación, Escuela Superior de Medicina, Instituto Politécnico Nacional, Mexico City C. P. 11340, Mexico; mgutierrezsa@ipn.mx (M.G.-S.);; 2 Laboratorio de Microbiología, Grupo CyMA, Unidad de Investigación Interdisciplinaria en Ciencias de la Salud y la Educación, UNAM FES Iztacala, Universidad Nacional Autónoma de México, Tlalnepantla de Baz C. P. 54090, Mexico; macaye@iztacala.unam.mx (M.M.C.-Y.);

**Keywords:** *Naegleria fowleri*, immunization, cholera toxin, nasal passages, nasal-associated lymphoid tissue

## Abstract

Background: *Naegleria fowleri* is a free-living amoeba that inhabits warm freshwater and causes primary amoebic meningoencephalitis (PAM), a rapidly fatal infection with >95% mortality. Due to the lack of early diagnosis and effective therapy, preventive vaccination represents a promising strategy. Methods: This study evaluated short- and long-term immune protection in BALB/c mice (20 mice per group) immunized intranasally with total *N. fowleri* extract co-administered with cholera toxin (CT). Mice were challenged with a lethal dose of trophozoites either 24 h (short-term) or three months (long-term) after the fourth immunization; the latter group received a booster 24 h before challenge. Serum and nasal washes were analyzed for IgA and IgG antibodies by immunoblot, and lymphocyte subsets from nasal-associated lymphoid tissue (NALT) and nasal passages (NPs) were characterized by flow cytometry. Results: Immunization conferred complete (100%) survival in the 24 h group and 60% protection in the 3-month group, whereas all control mice died. Immunoblotting showed that IgA and IgG antibodies recognized major *N. fowleri* antigens of 37, 45, 48 and 19, 37, and 100 kDa, respectively. Flow cytometry revealed increased activated and memory B lymphocytes, dendritic cells, and expression of CCR10, integrin α4β1, and FcγRIIB receptors, particularly in the 24 h group. Conclusions: Intranasal immunization with *N. fowleri* extract plus CT elicited both systemic and mucosal immune responses capable of short- and long-term protection. These findings highlight the potential of this immunization strategy as a foundation for developing effective vaccines against PAM.

## 1. Introduction

*Naegleria fowleri*, commonly known as the “brain-eating amoeba”, is a free-living protozoan found in warm aquatic environments, particularly in freshwater bodies and poorly chlorinated swimming pools. Infection occurs when contaminated water enters the nasal passages, allowing the amoeba to migrate along the olfactory nerves, cross the cribriform plate, and reach the central nervous system, where it triggers disease [1]. This amoeba is the etiological agent of primary amoebic meningoencephalitis (PAM), a fulminant CNS infection with a mortality rate exceeding 95% [2]. This pathology manifests as cerebral edema, hemorrhagic necrosis, brain herniation, and, in most cases, results in a fatal outcome [3]. Therapeutic options for PAM remain limited, as few rigorous clinical studies or controlled investigations are available [2]. Furthermore, there is currently no validated vaccine against *N. fowleri* available for human use [4].

In our working group, we aim to develop candidate vaccines against *N. fowleri* for potential use in humans. Our protection model involves immunizing BALB/c mice intranasally with an amebic extract of *N. fowleri*, combined with CT as an adjuvant. After four immunizations, the mice are challenged with the lethal dose of *N. fowleri* trophozoites.

The use of cholera toxin as an adjuvant is supported by its ability to strongly enhance mucosal and systemic immune responses. CT improves antigen uptake, promotes activation of B and T lymphocytes, and favors the induction of IgA responses, which are key for protection at mucosal surfaces [5,6,7]. For these reasons, CT has been widely used as an effective experimental mucosal adjuvant in preclinical models.

These immunostimulatory properties likely contribute to the efficacy observed in our model and explain, at least in part, the strong protective outcomes obtained. Indeed, immunized mice showed a 100% survival rate after challenge over a period of 60 days [8]. It has been proposed that the protection of immunized mice is due to the specific immune response against *N. fowleri* antigens, as well as to the participation of a Th2 profile response, together with an increase in the expression of genes such as the IgA alpha chain, the polymeric immunoglobulin receptor (pIgR) and cytokines such as IL-4, IL-6, IL-10 and IFN-γ. In addition, there is an increase in the secretion of IgA, IgG and IgG1 antibodies in mucosal membranes, which suggests that these antibodies prevent both adhesion and subsequent invasion of the amoeba to the nasal epithelium [8,9].

In another study, using the same immunization schedule mentioned above, T and B lymphocyte populations were examined. The results revealed a higher presence of CD4 T lymphocytes compared to CD8, in addition to an increase in the expression of CD45 on B cells, as well as IgA antibody-forming cells (IgA-AFCs), mainly in the nasal passage (NP) and nasopharyngeal-associated lymphoid tissue (NALT) regions [10]. We have also observed that polymorphonuclear cells (PMNs) present in the lumen are activated by their FcγRIII receptor, which recognizes the Fc fraction of antigen–antibody complexes. Once activated, these PMNs are capable of eliminating *N. fowleri* trophozoites [11]. Furthermore, secretory immunoglobulin A (sIgA), by binding to trophozoites, neutralizes their motility, preventing them from initiating the infectious process [9]. Furthermore, macrophages and dendritic cells from the NALT, NP, and cervical lymph nodes (CLN) in immunized mice express elevated levels of CD80 and CD86. This suggests that the increased specific immune response against *N. fowleri* antigens could be attributed to the immunization schedule used, based on total *N. fowleri* extracts co-administered with CT as an adjuvant, since both promote the migration and maturation of antigen-presenting cells (APCs), including macrophages (MQs) and dendritic cells (DCs) [10]. Taken together, all of these factors suggest that both innate and adaptive immunity play a crucial role in host protection against *N. fowleri* infection [12]. However, in the described protection model, the long-term immune response has not been evaluated, which is crucial for developing vaccine candidates that elicit robust and lasting immune responses.

Because immunological memory constitutes the basis of vaccination, allowing the immune system to respond more vigorously when encountering the same pathogen again [13], we hypothesized that intranasal immunization with an amoebal extract of *N. fowleri* co-administered with CT would (i) increase survival after lethal challenge in both short- (24 h) and long-term (3 months) immunized groups, (ii) induce significant local and systemic IgA and IgG responses in these groups, and (iii) modulate the effector and memory B lymphocyte populations over time.

## 2. Methodology

### 2.1. Animals

Male BALB/c mice, 8 to 10 weeks old, were used. All experiments were conducted in compliance with the Mexican Official Standard: NOM-062-ZOO-1999 “Technical specifications for the production, care, and use of laboratory animals” SAGARPA “Guide for the care and use of laboratory animals” [14] and approved by the Institutional Animal Care and Use Committee (ESM-CICUAL-ADEM-05/27-09-2019). The mice were kept in propylene cages, with 12 h light and dark cycles, and were provided with water and food *ad libitum*. The mice were acclimatized to laboratory conditions for one week prior to the experiments.

### 2.2. Amoebal Extract of N. fowleri

*N. fowleri* trophozoites (ATCC 30808) were maintained under axenic conditions at 37 °C in 2% Bactocasiton medium supplemented with 10% fetal bovine serum (FBS) and 0.1% penicillin-streptomycin. To obtain the extract, trophozoites were harvested at the logarithmic phase of growth (72 h) and centrifuged 1500× *g* for 10 min. Cells were washed twice with PBS-1x and resuspended in 1 mL of 5 mM p-hydroxy-mercurybenzoic acid (H-0642; Sigma-Aldrich Co., St. Louis, MO, USA) as a protease inhibitor. The amoebae were lysed by sonication (BRANSON Digital Sonifier model S-150D) with 10 s cycles at an amplitude of 100 W. The resulting extract was stored at −70 °C for later use. Protein concentration was determined by the Bradford technique, and the protein pattern was examined by SDS-PAGE electrophoresis.

### 2.3. Immunization Schedule

For the immunization schedule, groups of 20 mice were lightly anesthetized with ethyl ether and immunized intranasally with a mixture consisting of 100 µg of *N. fowleri* amoebal extract per dose (protein concentration 3.3 µg/µL) and 2 µg of cholera toxin (C8052; Sigma-Aldrich Co., St. Louis, MO, USA) per dose.

Mice were immunized at 7-day intervals on four occasions, on days 1, 7, 14, and 21. They were then divided into two groups. Group 1: mice challenged with 6 × 10^3^ *N. fowleri* trophozoites 24 h after the last immunization (24 h group); group 2: mice that received a booster (100 μg of *N. fowleri* amoeba extract of *N. fowleri* were mixed with 2 μg of CT) three months after the last immunization. 24 h after the booster, they were challenged with 6 × 10^3^ trophozoites (3-month group (3 M)). Control mice received only 30 μL of PBS. Ten mice from each group were monitored for 60 days to assess survival rates. The remaining ten mice from each group were sacrificed 24 h after challenge for immune response analysis. Serum and nasal washes (NW) samples, as well as nasal-associated lymphoid tissue (NALT) and nasal passages (NPs) cells, were obtained.

### 2.4. Obtaining Cells from NALT and NPs

The NALT was obtained by incising the palate along the inner contour of the teeth with a scalpel blade. The tissue was placed in a Petri dish with 2 mL of cold PBS-1x. The tissue was carefully disaggregated and filtered to recover the supernatant in a final volume of 2 mL of PBS-1x. The samples were centrifuged at 276× *g* for 5 min at 24 °C. Once the cell pellet was obtained, it was resuspended in 1 mL of PBS-1x.

To obtain NPs, the skin overlying the nose was removed and a cut was made below the eyes. The tissue was placed in 7.5 mL of RPMI with collagenase and Dichlorodiphenyltrichloroethane (DDT) to be disaggregated. The samples were then incubated at 37 °C for 30 min in a water bath, shaking vigorously every 5 min. After the incubation time, the samples were filtered and centrifuged for 5 min at 276× *g* at room temperature. Lymphocyte collection was performed using a Percoll gradient. The cell pellet obtained from the NPs was resuspended in 5 mL of 40% Percoll, which was slowly added to 5 mL of 70% Percoll to maintain the gradient. The samples were centrifuged at 595× *g* for 25 min at 24 °C without acceleration or deceleration. Lymphocytes were recovered from the interface formed between both gradients, which was washed with RPMI medium supplemented with FBS and centrifuged at 769× *g* for 5 min at 24 °C, the cell pellet was resuspended in 1 mL of PBS.

### 2.5. Western Blot

Protein separation was performed by SDS-PAGE (10%) of amoebal extract of *N. fowleri* (20 µg), and then the proteins were transferred to a nitrocellulose membrane using a power source applying an amperage of 400 mA/1 h. The membranes were blocked with 10% skim milk for 24 h. The strips were washed with PBS with Tween 20 (PBS-T) post incubation. Subsequently, primary antibodies from sera and nasal washes (1:100 and 1:1, respectively) from the control, 24 h, and 3 M mouse groups were applied and incubated overnight at 4 °C. The strips were washed three times with PBS-T. They were then incubated with the secondary antibody, anti-mouse IgG-PO and anti-mouse IgA-PO (1:6000, 1:500, respectively) overnight at 4 °C. After the incubation time, they were washed three times with PBS-T. Finally, the staining was developed by adding the solution (4-chloro-1-naphthol/methanol/hydrogen peroxide 30%) and washed with PBS-T three times.

### 2.6. Flow Cytometry

For phenotypic determination of cells from different tissues (NALT and NPs), cells were adjusted to 1 × 10^6^ in 200 µL of PBS-1x for each stain. The samples were centrifuged for 5 min at 276× *g* at 4 °C; the supernatant was decanted and 10 µL of the antibody cocktail was added, for effector B lymphocytes we used murine antibodies: anti-B220 peridinin-chlorophyll-protein complex (PerCP) (BioLegend, Inc., San Diego, CA, USA, #103234) and anti-CD138 phycoerythrin (PE) (BioLegend, Inc., San Diego, CA, USA, #142504); for Ab-secreting cells (ASCs): anti-CCR10 allophycocyanin (APC) (R&D Systems, Minneapolis, MN, USA, #FAB2815A), anti-IgA fluorescein isothiocyanate (FITC) (BioLegend, Inc., San Diego, CA, USA, #559354). On the other hand, for the preparation of the following antibodies for activated B cells: anti-B220/PerCP (BioLegend, Inc., San Diego, CA, USA, #405305), anti-CD138/APC (BioLegend, Inc., San Diego, CA, USA, #142506), anti-α4β1/PE (BioLegend, Inc., San Diego, CA, USA, #102206); for ASCs: anti-IgG/FITC. On the other hand, for memory B lymphocytes the antibodies used were anti-CD19/APC (BioLegend, Inc., San Diego, CA, USA, #550942), anti-CD80/PE (BioLegend, Inc., San Diego, CA, USA, #553769), for ASCs: anti-IgG/FITC and anti-IgA/FITC; for DCs CD11c/PerCP (BioLegend, Inc., San Diego, CA, USA, #117326) and FCγRIIB/PerCP (BioLegend, Inc., San Diego, CA, USA, #101324) were used. Subsequently, the samples were incubated for 20 min at room temperature. After the incubation time, the samples were resuspended in 200 μL of PBS-1x and centrifuged for 5 min at 276× *g* at 4 °C. Finally, the supernatant was decanted, and the cells were resuspended in 500 μL of 1% paraformaldehyde (PFA). For intracellular markers, the cells were fixed (Cytofix-Cytoperm, BD Biosciences, San Jose, CA, USA, #554722), permeabilized (Perm/Wash buffer, BD Biosciences, San Jose, CA, USA, # 554723) and stained according to the BD Bioscience protocol for intracellular staining. To perform relative fluorescence analysis, signal intensities were measured and analyzed using a MACSQuant analyzer 10 flow cytometer (Miltenyi Biotec, Bergisch Gladbach, Germany). Events were obtained by lymphocyte gating on the FSC/SSC dot plot. For each phenotypic trait, data for 20,000 events were obtained using MACSQuantify™ Software (Miltenyi Biotec, Bergisch Gladbach, Germany). Results represent the percentage of positively stained cells in the total cell population. Cell populations were analyzed using MACSQuantify™ v3.0.1 software. Data from 5 mice per group are expressed as mean ± SD.

### 2.7. Statistical Analysis

Statistical analysis of the data obtained was performed by multiple comparison between the control group and the immunized groups, by two-way ANOVA with a *p* < 0.05, *p* < 0.01 or *p* < 0.001, likewise the statistically significant difference in the percentage of cells between the groups was indicated as follows: (* *p* < 0.05; ** *p* < 0.01; *** *p* < 0.001) with respect to the control group, (^+^ *p* < 0.05, ^++^ *p* < 0.01 or ^+++^ *p* < 0.001) compared to the groups of mice of 24 h, 3 M and (ns) no statistically significant difference was found between the different groups studied, all analyzes were performed with the PRISM GraphPad v 9.4.1 program. For all the graphs reported in this work, the data represent the mean ± SD of three independent experiments.

Densitometric analysis of the Western blot strips was performed using ImageJ software, version 1.54h2 (Wayne Rasband/NIH, Bethesda, MD, USA, 2024), which allowed accurate and reproducible quantification of the immunoreactive bands detected by Western blot. In all gels, the Bio-Rad Precision Plus Protein Dual Color Standards (catalog #1610374) were used as the molecular weight marker to determine the approximate molecular weight of the immunoreactive bands observed.

## 3. Results

### 3.1. Evaluation of the Survival Rate of Immunized Mice

**Figure 1:**
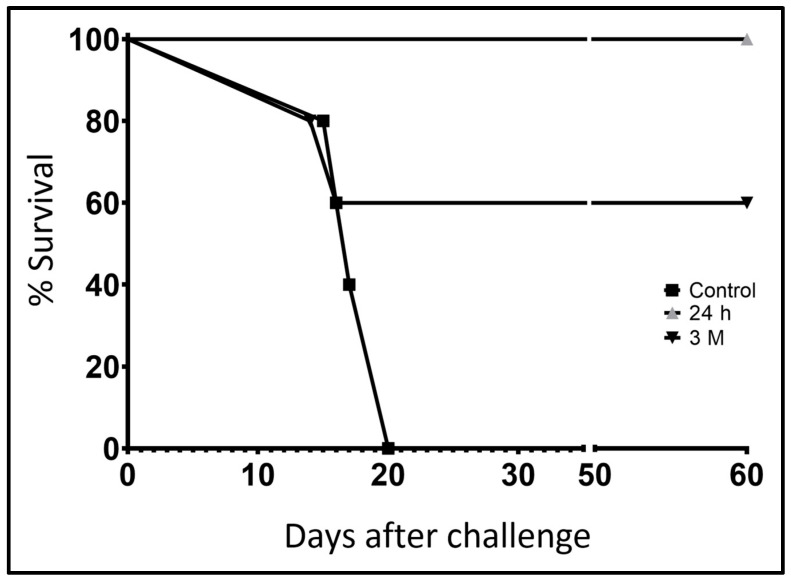
Survival percentage of BALB/c mice. Groups (*n* = 10 animals per group) of BALB/c mice were immunized i.n. with: Group 1: amoebal extract +CT 4 times every 7 days (Group 24 h, gray triangle), Group 2: amoebal extract +CT 4 times every 7 days and a booster immunization was administered three months later with amoebal extract +CT (3 M group, inverted black triangle) and the control group was only administered PBS (black square). All animals were challenged 24 h after the last immunization with 6 × 10^3^ *N. fowleri* trophozoites. Survival was recorded for 60 days and analyzed using the Log-rank (Mantel–Cox) test.

The results showed that control mice died between 16 and 20 days after infection with *N. fowleri* (Figure 1, black square). Furthermore, the 3 M group of mice had 60% protection against challenge with *N. fowleri* trophozoites (Figure 1, inverted black triangle). The 24 h group of mice had 100% survival (Figure 1, gray triangle).

### 3.2. Protein Recognition in Amoebal Extract of N. fowleri by the Western Blot Technique

**Figure 2:**
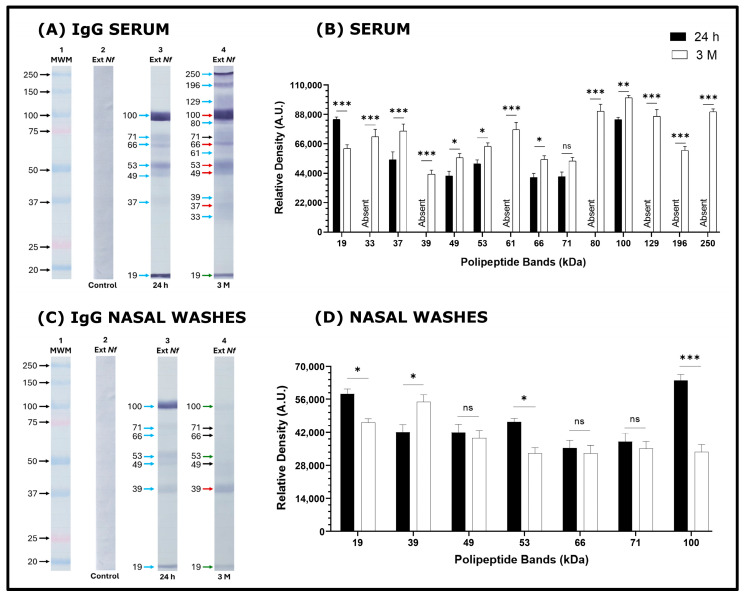
Recognition of bands from amoebal extract of *N. fowleri* by IgG antibodies from sera and nasal washes of immunized mouse groups by Western blot technique. (**A**) *N. fowleri* ET polypeptide bands recognized by IgG antibodies in (**A**) serum or (**C**) NW of the groups: control, strip 2 (**A**,**C**); 24 h, strip 3 (**A**,**C**) and 3 M, strip 4 (**A**,**C**). Molecular weight marker (MWM, strip 1 (**A**,**C**). Differences in the relative density of the bands were determined using Image J software (recognition of bands by IgG antibodies in: (**B**) Serum or (**D**) NW). Data represents the percentages of the mean SD of three independent experiments. The data obtained were statistically analyzed by means of an analysis of variance (ANOVA) and a Tukey multiple comparison test. A significance level of *p* < 0.05, *p* < 0.01 or *p* < 0.001 was considered to establish the existence of a significant difference between the bands compared * *p* < 0.05, ** *p* < 0.01, *** *p* < 0.001 and (ns) no statistically significant difference was found. Blue arrows indicate the band recognized for the first time. Red arrows show a statistically significant increase in band intensity in the 24 h group compared to 3 M, whereas black arrows indicate no significant difference between the 24 h and 3 M groups. Green arrows represent a statistically significant decrease in band intensity in the 24 h group compared to 3 M.

The recognition of the polypeptide bands in amoebal extract of *N. fowleri* was performed with serum samples and NW from the 24 h and 3-month-old groups of mice. Relative band intensity was quantified using ImageJ and normalized against the corresponding control to allow comparison among samples. The 19, 37, 49, 53, 66, 71, and 100 kDa bands were recognized by the IgG antibodies present in the serum samples of both immunized groups (Figure 2A,B). The intensity of recognition of the 19 and 100 kDa bands in the samples of the two immunized groups stands out. The 33, 39, 61, 80, 129, 196 and 250 kDa bands were recognized by IgG from serum samples of the 3 M group of mice. While IgG antibodies from NW samples of 24 h mice recognized the 19, 39, 49, 53, 66, 71 and 100 kDa bands. Standing out a higher intensity in the recognition of the 19, 53 and 100 kDa bands compared to the 3 M group (Figure 2C,D). NW IgG antibodies recognized the 19, 39, 49, 53, 66, 71, and 100 kDa bands in the 3 M group (Figure 2C,D). In this group, NW IgG recognized the 19, 53, and 100 kDa bands with lower intensity compared to the 24 h group. Only the 39 kDa band was recognized with greater intensity by the 3 M group compared to the 24 h group (Figure 2C,D).

**Figure 3:**
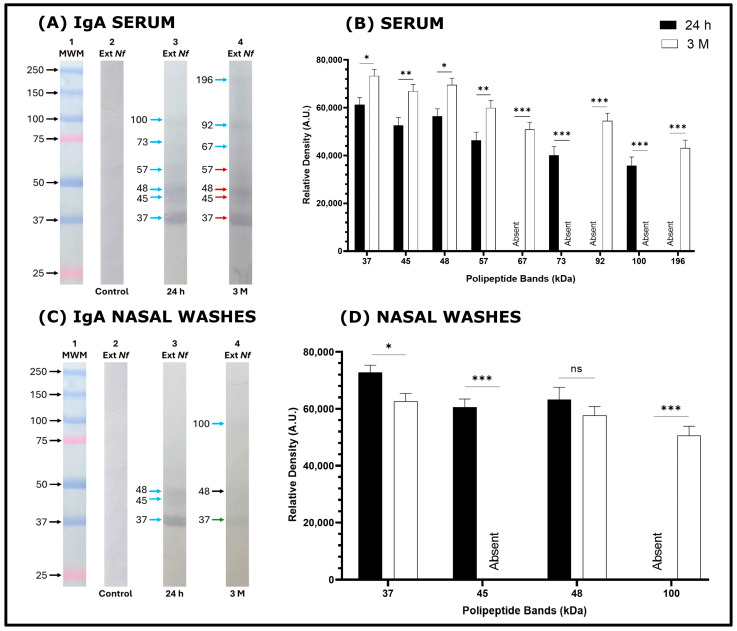
Recognition of bands from amoebal extract of *N. fowleri* by IgA antibodies from sera and NW of immunized mouse groups by Western blot technique. (**A**) amoebal extract polypeptide bands recognized by IgA antibodies in (**A**) serum or (**C**) NW of the following groups: control, strip 2 (**A**,**C**); 24 h, strip 3 (**A**,**C**) and 3 M, strip 4 (**A**,**C**). Molecular weight marker (MWM, strip 1 (**A**,**C**)). Differences in the relative density of the bands were determined using Image J software (recognition of bands by IgA antibodies in: (**B**) Serum or (**D**) Nasal washes). Data represents percentages of the mean SD of three independent experiments. Data obtained were statistically analyzed by means of an analysis of variance (ANOVA) and a Tukey multiple comparison test. A significance level of *p* < 0.05, *p* < 0.01 or *p* < 0.001 was considered to establish the existence of a significant difference between the bands compared * *p* < 0.05, ** *p* < 0.01, *** *p* < 0.001 and (ns) no statistically significant difference was found. Blue arrows indicate the band recognized for the first time. Red arrows show a statistically significant increase in band intensity in the 24 h group compared to 3 M, whereas black arrows indicate no significant difference between the 24 h and 3 M groups. Green arrows represent a statistically significant decrease in band intensity in the 24 h group compared to 3 M.

Fewer bands were recognized from the amoebal extract of *N. fowleri* by IgA antibodies in serum and NW samples, and those recognized had lower intensity. (Figure 3A,D). The serum IgA from the 3 M group recognized the 37, 45, 48, and 57 kDa bands with greater intensity than the 24 h group (Figure 3A,B). The 73 kDa and 100 kDa bands were more intense in the 24 h group compared to the 3-month group. (Figure 3A,B). The 73 kDa band was recognized by serum IgA in the 24 h group but was absent in the 3 M group. Whereas the 92 and 196 kDa bands were recognized by serum IgA antibodies in the 3 M group and were absent in the 24 h group. Additionally, the recognition of the bands by NW IgA antibodies was much lower than that of serum IgA (Figure 3C). The 37 and 48 kDa bands were recognized in both groups of immunized mice. The 37 kDa band was recognized with greater intensity in the 24 h group compared to the 3 M group, while the 48 kDa band was recognized with equal intensity in both groups of immunized mice. The 45 kDa band was recognized only in the 24 h group, and the 100 kDa band was recognized only in the 3 M group.

### 3.3. Phenotypic Analysis of Effector or Memory B Lymphocytes in the 24 h and 3 M Groups of Mice in NALT and NPs

**Figure 4:**
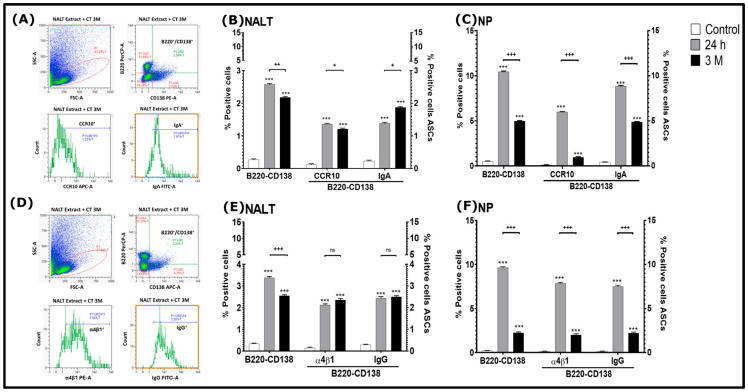
Phenotypic analysis of B220^+^-CD138^+^ effector B cells expressing CCR10^+^, α4β1^+^, IgA^+^ and IgG^+^ in NALT and NPs. From BALB/c mice groups of: (1) 24 h, (2) 3 M or control (only PBS was administered), NALT and NP cells were suspended for analysis by flow cytometry. (**A**,**D**) Representative dot-plots and histograms for cell population selection criteria for NALT tissue from the 3 M (**A**) or 24 h (**D**) group. Population selection was based on granularity and size using FSC-A and SSC-A MACSQuantify™ Software, version 3.1 (Miltenyi Biotec, Bergisch Gladbach, Germany) that determines the lymphocyte region; the same process was performed in all tissues from the different groups of mice. The percentage of cells positive for NALT (**B**,**E**) and NPs (**C**,**F**) of the different antibodies used is shown for cells positive for: B220^+^, CD138^+^, CCR10^+^ and IgA^+^, (**B**,**C**) and for: B220^+^, CD138^+^, α4β1^+^ and IgG^+^. The data obtained were statistically analyzed by analysis of variance (ANOVA) and a Tukey post hoc test. The significant difference (*p* < 0.001) of the percentage of cells between the groups is indicated as follows: *** *p* < 0.001 with respect to the control group; (^+^ *p* < 0.05, ^++^ *p* < 0.01 or ^+++^ *p* <0.001) comparison between groups: 24 h, 3 M and (ns) no statistically significant difference was found.

**Figure 5:**
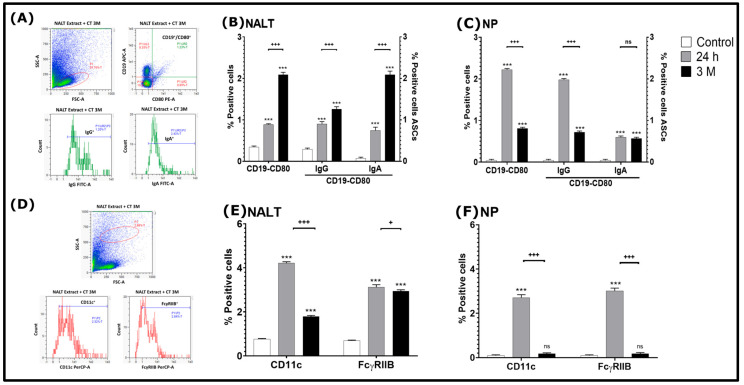
Phenotypic analysis of IgA^+^ and IgG^+^ memory B cells and DCs in NALT. From BALB/c mice groups of: (1) 24 h, (2) 3 M or control (only PBS was administered), NALT cells were suspended for analysis by flow cytometry. (**A**,**D**) Representative dot-plots and histograms for cell population selection criteria for NALT tissue from the 3 M group. Population selection was based on granularity and size using FSC-A and SSC-A MACSQuantify™ Software, version 3.1 (Miltenyi Biotec, Bergisch Gladbach, Germany) that determines the lymphocyte region, the same process was performed in all tissues from the different groups of mice. Population selection was based on granularity and size using the FSC-A and SSC-A programs that determine the lymphocyte region. The same process was performed in all tissues and at different times. The percentage of cells positive for NALT (**B**) and NPs (**C**) of the different antibodies used is shown for memory B cells: CD19^+^, CD80^+^ and ASCs: IgG^+^ and IgA^+^; NALT (**E**) and NP (**F**) for DCs: CD11c^+^ and FcγRIIB^+^. The data obtained were statistically analyzed by analysis of variance (ANOVA) and a Tukey post hoc test. The significant difference (*p* < 0.001) of the percentage of cells between the groups is indicated as follows: *** *p* < 0.001 with respect to the control group; (^+^ *p* < 0.05 or ^+++^ *p*< 0.001) comparison between the groups of: 24 h and 3 M and (ns) no statistically significant difference was found.

The percentage of effector B cells in NALT (Figure 4 A,B,D,E) or NP (Figure 4C–F), as well as memory B cells in the NALT (Figure 5A,B) and DCs (Figure 5C,D) of control or immunized mice was analyzed by flow cytometry. In the case of NALT, a statistically significant increase in the percentage of activated B220^+^/CD138^+^ B cells was observed in all immunized groups compared to the control group (*p* < 0.001) (Figure 4B). However, there was a statistically significant decrease in B220^+^/CD138^+^ B cells in the 3 M group compared to the 24 h group (*p* < 0.01) (Figure 4B). In the case of ASCs that expressed CCR10^+^, a statistically significant increase was observed in all immunized groups compared to the control group (*p* < 0.001). However, there was a statistically significant decrease in CCR10^+^ in the 3 M group compared to the 24 h mice (*p* < 0.05) (Figure 4B). Regarding ASCs that expressed IgA^+^, a statistically significant increase was observed in all immunized groups compared to the control group (*p* < 0.001). However, there was an increase in IgA^+^ in the 3 M group (1.85 ± 0.057%) compared to the 24 h group (1.37 ± 0.075%) (*p* < 0.05 respectively) (Figure 4B).

In NPs, there was a statistically significant increase in CCR10^+^ and IgA^+^ in all immunized groups compared to the control group (*p* < 0.001) (Figure 4C). However, there was a statistically significant decrease in B220^+^/CD138^+^ B lymphocytes, as well as ASCs expressing CCR10^+^ and IgA^+^ in the 3 M group compared to the 24 h group (*p* < 0.001) (Figure 4C).

On the other hand, in the NALT, a statistically significant increase in the percentage of B220^+^/CD138^+^ B lymphocytes, as well as in the expression of integrin α4β1^+^ and IgG^+^, was observed in all immunized groups compared to the control group (*p* < 0.001) (Figure 4E). Likewise, a decrease in the percentage of B220^+^/CD138^+^ B lymphocytes was observed in 3 M mice (2.16 ± 0.071) compared to the 24 h group (*p* < 0.001). However, regarding cells positive for α4β1^+^ and IgG^+^, these did not have a statistically significant difference between the immunized groups (Figure 4E).

On the other hand, a statistically significant increase in the percentage of α4β1^+^ and IgG^+^ cells was found in NPs in all immunized groups compared to the control group (*p* < 0.001) (Figure 4F). However, a statistically significant decrease in these cells was found in the 3 M Group compared to the 24 h group (*p* < 0.001) (Figure 4F).

In both NALT and NPs (Figure 5A–C), a statistically significant increase in CD19^+^/CD80^+^ memory B cells, as well as IgG^+^ and IgA^+^ expressing ASCs, was observed in all immunized groups compared to the control group (*p* < 0.001). Additionally, in NALT, the 3 M group had an increase in these cells compared to the 24 h group (*p* < 0.001) (Figure 5B). However, in NPs, a significant increase in CD19^+^/CD80^+^ memory B cells, as well as IgG^+^ expressing ASCs, was found in the 24 h group compared to the 3 M group (*p* < 0.001); however, in IgA-expressing cells, no statistically significant difference was found between the immunized groups (*p* < 0.01) (Figure 5C). In this same sense in NALT and in NPs there was a statistically significant increase in CD11c^+^ and FcγRIIB^+^ DCs in all immunized groups compared to the control group (*p* < 0.001), except for the 3 M group in NPs which had no statistically significant difference in these mentioned cells with respect to the control group (Figure 5D–F); however, in both NALT and NPs, there was a statistically significant decrease in CD11c^+^ and FcγRIIB^+^ DCs in the 3 M group compared to the 24 h group (*p* < 0.001 and *p* < 0.05) (Figure 5E,F).

## 4. Discussion

The objective of this study was to analyze the protective immune response in BALB/c mice immunized intranasally on four occasions with amoebal extract of *N. fowleri* co-administered with CT. Survival was measured 24 h and 3 months after the last immunization. The 3 M group received a booster of amoebal extract of *N. fowleri* plus CT 24 h before the challenge. The immunization schedule used induced 100% protection in the 24 h mice and 60% protection in the 3 M mice. These results suggest that the efficacy of inducing long-term protection in the *N. fowleri* meningitis model depends on the immunization schedule used, as well as the immune factors activated by the immunization.

In a previous work [8], we reported that intranasal immunization of BALB/c mice with an amoebic extract of *N. fowleri* combined with cholera toxin (CT) administered four times at seven-day intervals, followed by a challenge 24 h after the last immunization, resulting in 100% protection for the mice. This immunization also elicited both local and systemic IgG and IgA antibody responses. The immunoblot technique revealed a greater recognition of *N. fowleri* antigens by IgG antibodies from both serum and nasal wash (NW) samples compared to the recognition of antigens by IgA antibodies in those samples. In the present study, the response observed in the immunoblots performed with serum and NW samples from 24 h and 3 M mice was quite similar. It was noted that IgG antibodies recognized a larger number of bands from *N. fowleri* extracts in both serum and NW samples compared to the bands recognized by IgA from the same samples. Additionally, IgG detected more bands than IgA in either serum or NW (Figure 2 and Figure 3). However, Carrasco-Yépez et al. [9] using the immunohistochemistry technique detected high levels of both IgA and IgG in the lamina propria, epithelial cells and in the lumen of the olfactory mucosa in mice that were immunized according to the aforementioned schedule. The results indicate that the low recognition of bands by IgA and IgG in NW is primarily due to the very low recovery of antibodies when these washes are performed using PBS, compared to their concentrations in serum. Considering this, we observed that serum IgG antibodies from the 24 h and 3 M groups, as well as IgG from NW from the 24 h group, strongly recognized the 100, 66, 57, 50, 37, and 19 kDa bands in the *N. fowleri* extracts. This observation aligns with the immunization schedule previously reported. [8]. The IgA from NW samples in the 24 h and 3 M groups showed recognition of some of these bands, although with lower intensity. This observation highlights that in the 3 M group, there was less recognition of bands, and the bands that were recognized were identified with reduced intensity. The lower band recognition in the 3 M group would be associated with the 60% protection achieved in this group. The role of antibodies in protection was reported by Rojas-Ortega et al. [11] who reported on the role of antibodies in providing protection against *N. fowleri*. They propose that PMNs are activated via their FcγRIII receptors when they encounter antigen–antibody complexes in the lumens of challenged mice. This mechanism suggests that both PMNs and the antibodies IgG and IgA play a crucial role in the defense against *N. fowleri*.

Although our results emphasize the relevance of mucosal IgA, the Western blot data clearly show that IgG recognition dominates over IgA in both serum and NW samples. This pattern is a well-established consequence of humoral compartmentalization: IgG is abundant in serum and can transudate into mucosal secretions, especially after repeated immunization or local inflammation [15,16], making it highly detectable in immunoblot assays. In contrast, secretory IgA is produced locally and recovered at lower concentration in lavage samples, which can result in weak or limited band visualization, despite its essential effector role at mucosal surfaces [17]. IgG responses typically exhibit high affinity for their specific antigens as a result of germinal-center maturation [5,18], further reinforcing its apparent dominance in this type of assays. Therefore, the reduced IgA band recognition should not be interpreted as evidence of a minor protective contribution, instead, it is a result of the physiological distribution and the sensitivity of the methodologies used. In light of the strong IgG responses and cellular activation observed, it is reasonable to consider that IgG and other adaptive mechanisms may also play an important role in protection against *N. fowleri*, emphasizing the need for future functional studies to clarify the contributions of each immune component.

Furthermore, in the flow cytometry phenotypic analyses we performed in this study, we analyzed the expression of CD138, which is commonly recognized as a characteristic marker of plasma cells [19]. In addition, we evaluated the expression of B220, an isoform of CD45, which is considered a widely recognized marker in mouse B cells. This marker is present from the early stages of development of these cells and remains even in mature B cells after they have been exposed to an antigen, transit through the germinal centers (GCs) and have integrated into the memory B cell reservoir [20]. Concerning this, in this work, cells expressing these mentioned cellular markers (B220^+^/CD138^+^) were found to be increased in the 24 h group in both NALT and PN compared to the decrease seen in the 3 M group, In addition, the B220^+^/CD138-positive cells were also positive for IgA, so these cells were found in greater numbers compared to the control in these tissues and an increase in NPs compared to the 3 M group. (Figure 4B).

These results reinforce the protective immunity previously reported against *N. fowleri*, in which it has been found that the antigens of lysates of the amoeba and CT, when captured by APCs, trigger a key interaction with T cells located in the NPs and cervical lymph nodes (CN) regions. Subsequently these CD4^+^ cells stimulate the differentiation of B lymphocytes into IgA-producing cells [10]. This justifies in this work the greater protection obtained in the 24 h group of 100% compared to the decrease that was observed in the 3 M group with 60% (Figure 1).

The decline in long-term protection may be associated with the waning of mucosal IgA responses or with insufficient persistence of IgA-committed memory B cells, phenomena that have been described in the context of mucosal vaccine durability [5]. In accordance with this, the 24 h group not only presented a higher percentage of effector B lymphocytes but also showed an increase in IgA^+^ ASC in the nasal cavity (Figure 4B,C). Therefore, the enhanced presence of IgA antibodies could directly interact with trophozoites in the nasal lumen, blocking their ability to adhere to the epithelium and exerting a protective effect. Together, these observations underscore the central importance of the sIgA-mediated immune response in defense against this pathogen [5,9].

Furthermore, in this study, we also investigated the expression of the homing molecule CCR10. It is known that the migration of cells to mucosal tissues relies on the expression of CCR9 and CCR10 in plasma cells, along with their respective ligands, CCL25 and CCL28, which are expressed in mucosal tissues and endothelial cells. Flow cytometry analysis has revealed that plasma cells obtained from the NALT of immunized mice express CCR10, while exhibiting nearly undetectable levels of CCR9 [21]. Additionally, it was observed that IgA ASCs show a selective and preferential expression of the CCR10 receptor [22]. Therefore, we focused our study on CCR10 in the nasal cavity. Our findings revealed an increase in ASCs expressing the CCR10 homing molecule in the 24 h group compared to the 3 M group, both in the NALT and NP. (Figure 4B,C). Notably, the heightened expression of CCR10^+^ in pathogen-specific IgA ASCs in the 24 h group, which achieved 100% survival, could be a critical factor in establishing effective IgA-mediated immunity localized in mucosal membranes [23], compared to the 3 M group which exhibited a decrease in protection, with only 60% survival.

In this study, we analyzed the expression of integrin α4β1, which is commonly expressed in most lymphocytes, ranging from moderate to high levels, as well as in eosinophils and monocytes. This integrin acts as a receptor for various ligands, including vascular cell adhesion molecule 1 (VCAM-1), which is part of the immunoglobulin family, fibronectin, and the α4 subunit of integrin [24]. We observed a change in the percentage of B-lymphocyte positive (B220^+^/CD138^+^) expressing α4β1^+^ and IgG in NALT and NPs of the immunized groups. Although there was no significant difference in the percentages of these cells in the NALT, a higher percentage was observed in the 24 h NPs group of mice compared to the 3 M group (Figure 4E,F). Previous research has shown an increase in CD45^+^CD19^+^ B lymphocytes in mice immunized with *N. fowleri* lysates alone, those co-administered with cholera toxin (CT), and those that received CT alone, when compared to the untreated group [10]. In the case of B lymphocytes from the group immunized exclusively with CT, a significant increase in B220^+^/α4β1^+^ cells was recorded, both in the NALT and in the NPs, compared to the control group [12]. In this context, it was reported that immunization of BALB/c mice with *N. fowleri* lysates co-administered with CT or Cry1Ac as adjuvants induces a Th2-type immune response, characterized by an increase in the production of antibodies, specifically IgA and IgG [8,9]. In another study, Gutierrez-Sánchez et al. [12] evaluated the efficacy of an immunogenic peptide designed in silico from the *N. fowleri* membrane protein MP2CL5 (Smp145). The researchers immunized BALB/c mice with this peptide, administered alone or co-administered with CT, and then challenged with the lethal dose of *N. fowleri* trophozoites. The results demonstrated that the peptide induced 60% protection when administered alone and 80% when co-administered with CT. Immunization with both antigens (Smp145 alone or co-administered with CT) generated an increase in the population of T and B lymphocytes. Likewise, a higher level of expression of integrin α4β1 was detected in the protected mice, as well as an increase in IgA and IgG antibodies in the nasal cavity, suggesting a localized immune response. Therefore, in this study, the elevated survival rate observed in the 24 h group compared to the 3 M group suggests greater activation of an adaptive response against protective antigens. This mechanism could enhance the action of the innate immune components as neutrophils responsible in conjunction with the increased protection found in the 24 h group against *N. fowleri* compared to 3 M group [11].

In the present study, we also found an increase in memory cells (CD19^+^/CD80^+^) IgG^+^ and IgA^+^ in the immunized groups (Figure 5B,C). Consequently, it is important to mention that CD19 is crucial for regulating the activation, proliferation, and differentiation of B lymphocytes [25], while CD80 is a key marker of B cell activation, and its expression is associated with differentiation into memory B cells within GCs [26]. In relation to this, in this work it was found that B cells (CD19^+^/CD80^+^) had an increase in the NALT in the 3 M group (Figure 5B), while in the NPs the increase occurred in the 24 h group (Figure 5C). The differences in the distribution of these cells from the immunized groups between both tissues could be due, as previously proposed, to the migration of lymphocytes from the NALT to effector sites such as NPs, CN and distal organs such as the spleen (SP), which has been reported in previous studies with mice immunized by the i.n. route with *N. fowleri* antigens, either alone or co-administered with CT [10]. Furthermore, it is important to mention that NALT and local draining lymph nodes have the capacity to maintain long-term immunological memory. Isotype switching toward IgA, along with differentiation and maturation to ASCs, can be completed within the NALT itself before these cells move to other areas [27]. However, activated IgA-expressing cells in the CN could move to the NALT, NPs, the lamina propria of the nasal cavities, or even reach other distant mucosae, including the upper and lower respiratory tract, as well as the gastric and genital mucosae [5]. ASC-mediated IgG immune responses are dependent on these cells circulating through the draining lymph nodes of the NALT [27]. Therefore, taking all this together, we suggest that the higher survival rate observed in the 24 h group could be related to the rapid activation of memory B cells (CD19^+^/CD80^+^) and migration as plasmablast to the NPs where they complete their differentiation into plasma cells, and exert immediate effector functions through the production of IgA and IgG, which could translate into greater protection against *N. fowleri* challenge compared to the 3 M group. In addition, the early localization of memory B cells in NPs appears to be decisive for the protection and higher survival observed in mice in the 24 h group.

Additionally, the results of this study further showed a correlation between a higher percentage of CD11c^+^ and FcγRIIB^+^ cells in NALT and NPs with a higher survival rate in the 24 h group compared to the 3 M group (Figure 5E,F). Since CD11c is a widely recognized marker for identifying conventional DCs [28], these findings suggest that these cells could play a crucial role in the defense against *N. fowleri* in the 24 h group compared with 3 M group. In this regard, previous work has reported that following immunization with antigens (*N. fowleri* lysates co-administered with CT), APCs capture, process, and present these antigens to T lymphocytes in the NALT and CN. This mechanism could trigger the coordinated activation of T and B cells, inducing a robust immune response. As a consequence, it would increase the production of effector molecules capable not only of eliminating *N. fowleri*, but also of interfering with its adhesion to the nasal mucosa. In addition, in mice immunized with lysates of *N. fowleri* and CT, it has been found that in DCs obtained from NALT, the expression of CD86 has been found to be higher compared to that of CD80 [10]. CD86, together with CD80, is expressed on antigen-presenting cells, including B lymphocytes, and engages CD28 on T cells to provide essential costimulatory signals that support T cell activation [29,30]. The subsequent interaction of CD40L expressed on activated T cells with CD40 on B cells delivers critical signals required for B cell proliferation, survival, and initiation of differentiation [31]. In addition, when CD40 signaling is complemented by T cell-derived cytokines such as interleukin-21 (IL-21), B cells upregulate the transcription factor Blimp-1, promoting their differentiation into plasmablasts and ultimately plasma cells [32]. This mechanism is likely related to the observed increase in the IgA and IgG antibody response in mice immunized intranasally with *N. fowleri* and CT [8,9].

On the other hand, in this work, the higher percentage of FcγRIIB^+^ cells in the 24 h mice that had a higher percentage of protection could be due to the fact that this receptor is essential for GCs B cells capture antigens by follicular dendritic cells (FDC) through the its receptor that binds to the Fc region of the FcγRIIB antigen complexes, allowing affinity maturation and the selection of high-specificity B cells in collaboration with Tfh cells. This mechanism underlines the importance of the interaction between B cells, FDCs, and Tfh in the adaptive immune response, which is essential for generating long-term protection [33]. Given the above, the higher percentage of FcγRIIB^+^ cells in the 24 h group could favor a more efficient response in the GCs, allowing for faster and more specific maturation of B cells. This could contribute to more effective protection against challenge with *N. fowleri* infection.

## 5. Conclusions

Intranasal immunization with total *N. fowleri* extract co-administered with cholera toxin generated robust mucosal and systemic immunity, characterized by elevated IgA/IgG antibodies, memory B cells, and dendritic cell activation. The 100% short-term protection and 60% sustained protection observed after three months indicate strong but declining immunity over time. These results suggest that booster doses or refined antigen formulations may be required to achieve durable protection. Overall, this immunization model provides a promising experimental framework for future vaccine development against primary amoebic meningoencephalitis.

Intranasal immunization with *N. fowleri* extract plus cholera toxin elicited both short- and long-term protection in BALB/c mice, mediated by IgA/IgG antibodies, memory B cells, and dendritic cell activation. However, the decline in long-term protection emphasizes the need for booster optimization.

## Data Availability

Link to access the data that supports the results of this study: http://bit.ly/498ENh1, accessed on 25 October 2025.
